# Utilizing the novel AI-guided STAR Apollo™ Mapping System in complex redo-ablations: a case report of successful catheter ablation in a patient with persistent left superior vena cava

**DOI:** 10.1093/ehjcr/ytaf576

**Published:** 2025-11-08

**Authors:** Aaditya Vora, Tavleen Wasan, Matthew McKillop

**Affiliations:** Baptist Heart Hospital, 800 Prudential Drive, Jacksonville, FL 32207, USA; Imperial College London, Imperial College School of Medicine, Reynold Building, St Dunstan’s Road, London W6 8RP, UK; Baptist Heart Hospital, 800 Prudential Drive, Jacksonville, FL 32207, USA

**Keywords:** Case report, Atrial fibrillation, Electrophysiological mapping, Catheter ablation

## Abstract

**Background:**

Persistent left superior vena cava (PLSVC) can both initiate and perpetuate atrial fibrillation (AF) and poses several challenges to catheter ablation procedures. Novel mapping technologies offer promising potential to these complex patients. We present the first recorded case of artificial intelligence guided pulsed field ablation (PFA) in persistent AF with PLSVC.

**Case summary:**

A 70-year-old patient with PLSVC and persistent AF underwent a third-repeat catheter ablation. A three-dimensional geometry of the atria was generated, and STAR Apollo Mapping was used to identify target substrates [early sites of activation (ESAs) and repetitive pattern of activation STARbursts] in real time. Two ESAs were identified in the left atrium (LA) and three in the right atrium; all of these were targeted. Ablation in the PLSVC that overlaid an LA septal ESA terminated AF to sinus rhythm. Remapping confirmed pulmonary vein isolation and exit block, and no further arrhythmias were inducible. There were no procedural complications; the patient was discharged the same day.

**Discussion:**

STAR mapping aided identification of alternative ablation targets that would be challenging to identify using classical electro-anatomical mapping alone. This case demonstrates a novel use of this technology in improving procedural outcomes for complex AF patients.

Learning points

- Persistent left superior vena cava (PLSVC) can act as a trigger or driver of atrial fibrillation (AF) and poses several challenges to catheter ablation procedures. Successful identification of extra-pulmonary targets is important in improving outcomes in these patients, especially those undergoing repeat procedures.
- The STAR Apollo Mapping System is an AI-guided mapping technology that identifies target substrates regardless of AF mechanism; it was used with favourable outcomes in a highly complex case of recurrent AF with PLSVC and several other comorbidities.

## Introduction

Persistent left superior vena cava (PLSVC), the most common thoracic venous anomaly, can contribute to the onset and perpetuation of atrial fibrillation (AF).^1^ Persistent left superior vena cava prevalence is ∼0.3% in the general population, rising to 0.9% in AF patients and up to 11% in those with congenital heart disease.^2^ Persistent left superior vena cava has been indicated as a trigger or driver of AF in a majority of patients with both conditions.^3,4^ However, PLSVC poses several challenges for ablation.^5^ While studies suggest it may act as an arrhythmogenic source, evidence for successful PLSVC isolation in persistent AF using radiofrequency (RF) is limited, and only few case reports exist using pulsed field ablation (PFA).^6,7^

STAR Apollo Mapping (STAR Apollo Mapping System™, Rhythm AI Inc., CA) is a novel mapping system that uses artificial intelligence (AI)-guided technology to identify origins of AF regardless of underlying mechanisms. STAR Mapping underwent extensive development and validation in a series of single-centre studies; early results using radiofrequency energy to target identified sites showed improved long-term arrhythmic outcomes in patients with persistent AF.^8–11^ The accurate identification of extra-pulmonary target substrates plays an increasingly important role in improving ablation outcomes in complex patients such as those with PLSVC.^12^ We present the first case of AI-guided PFA for persistent AF with PLSVC, employing STAR Apollo Mapping in a patient undergoing a third-repeat catheter ablation. This marks the first use of the STAR Apollo Mapping System within the PLSVC. The data underlying this article are available in the article.

## Summary figure

**Figure:**
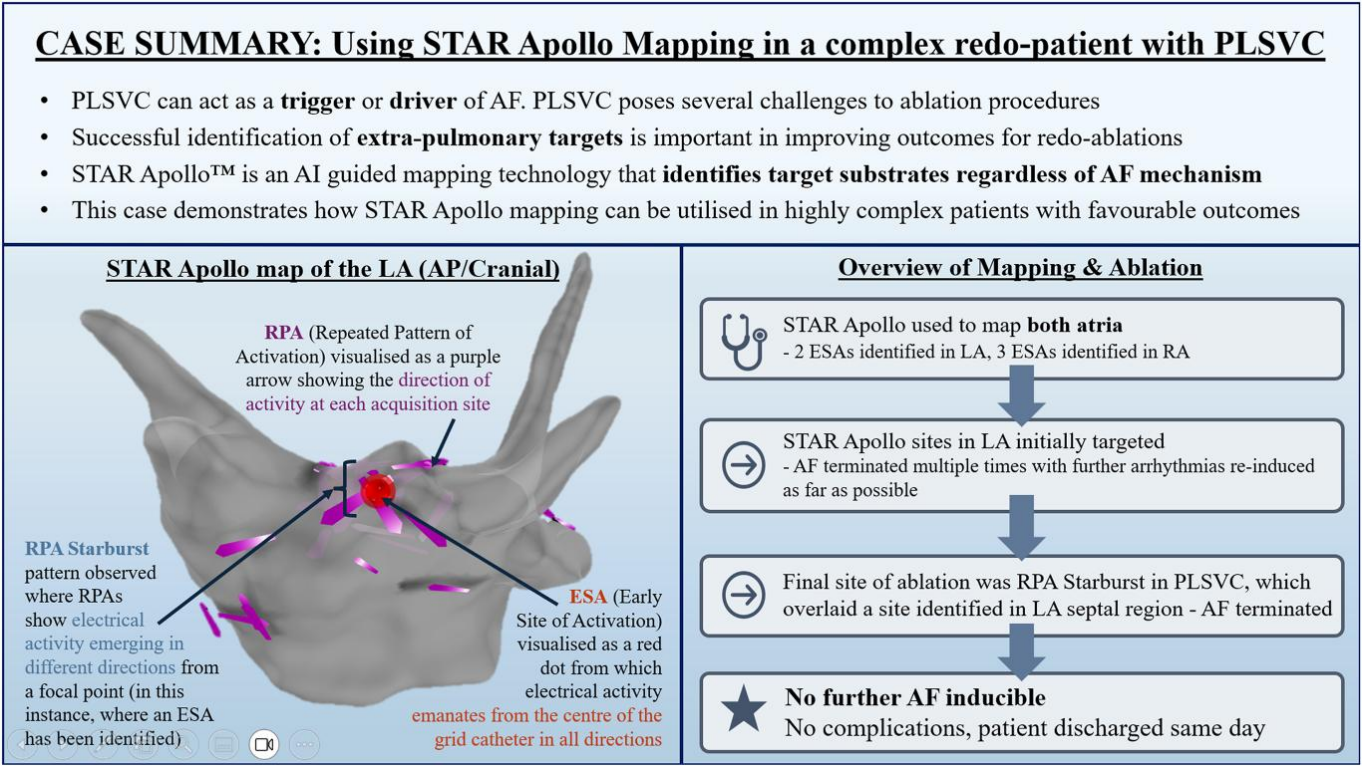


## Case presentation

A 70-year-old female patient with duplicate SVC with a PLSVC and persistent AF had previously undergone a surgical aortic valve replacement with concomitant pulmonary vein isolation, two previous catheter ablations (cryoballoon pulmonary vein isolation (PVI) and subsequent redo-PVI, posterior wall isolation, and fractionation targeting using radiofrequency), and coronary stenting. The existence of PLSVC had been discovered following the placement of a peripheral line, which demonstrated the course on fluoroscopy. Despite near-complete amelioration of symptoms with reestablishment of sinus rhythm, she re-presented with symptomatic palpitations, limiting fatigue, and shortness of breath. This temporally coincided with AF recurrence. Following discussion of other options (including pacemaker or further medical therapy), the patient elected to undergo a third catheter ablation. The procedure was performed under general anaesthetic with uninterrupted anticoagulation. Groin access was via the right femoral vein; intracardiac echocardiography was used to guide transseptal puncture.

### Mapping

A geometry of the atria and PLSVC was created using an 8 F HDGrid through a deflectable sheath (Abbott Inc., MN); catheter access to the PLSVC was via the large coronary sinus. Sequential 30-s electrogram recordings were performed and analysed in real time. A simultaneous voltage map was collected on the EnSite X mapping system (Abbott Inc., MN).

**Figure 1:**
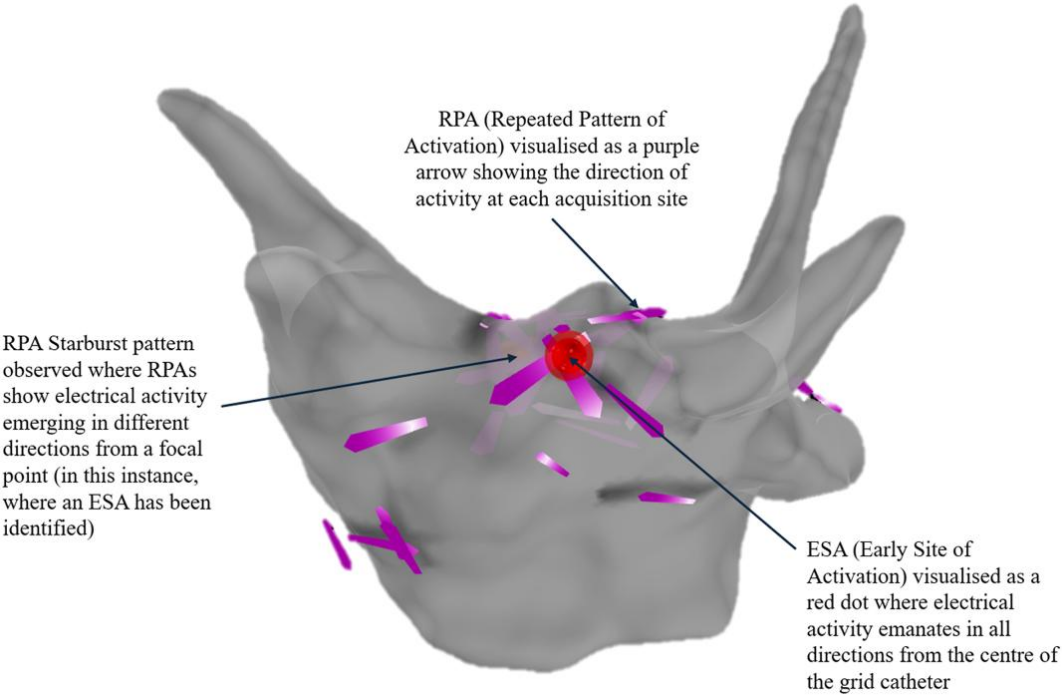
An example STAR Apollo map of the left atrium (AP/cranial view) with explanations of the sites identified.

The STAR Apollo Mapping System allows real-time data acquisition, analysis, and visualization by streaming electrogram data from the EnSite X system (Abbott) via the LIVESYNC module (Abbott). Early sites of activation (ESAs) are identified where electrical activity emanates in all directions from the centre of the catheter grid, suggesting a focal arrhythmogenic origin. Repetitive patterns of activation (RPAs) demonstrate the predominant activation; a ‘STARburst’ pattern (in which RPAs indicate activation away from a region) also acts as a site of interest (*Figure 1*).

**Figure 2:**
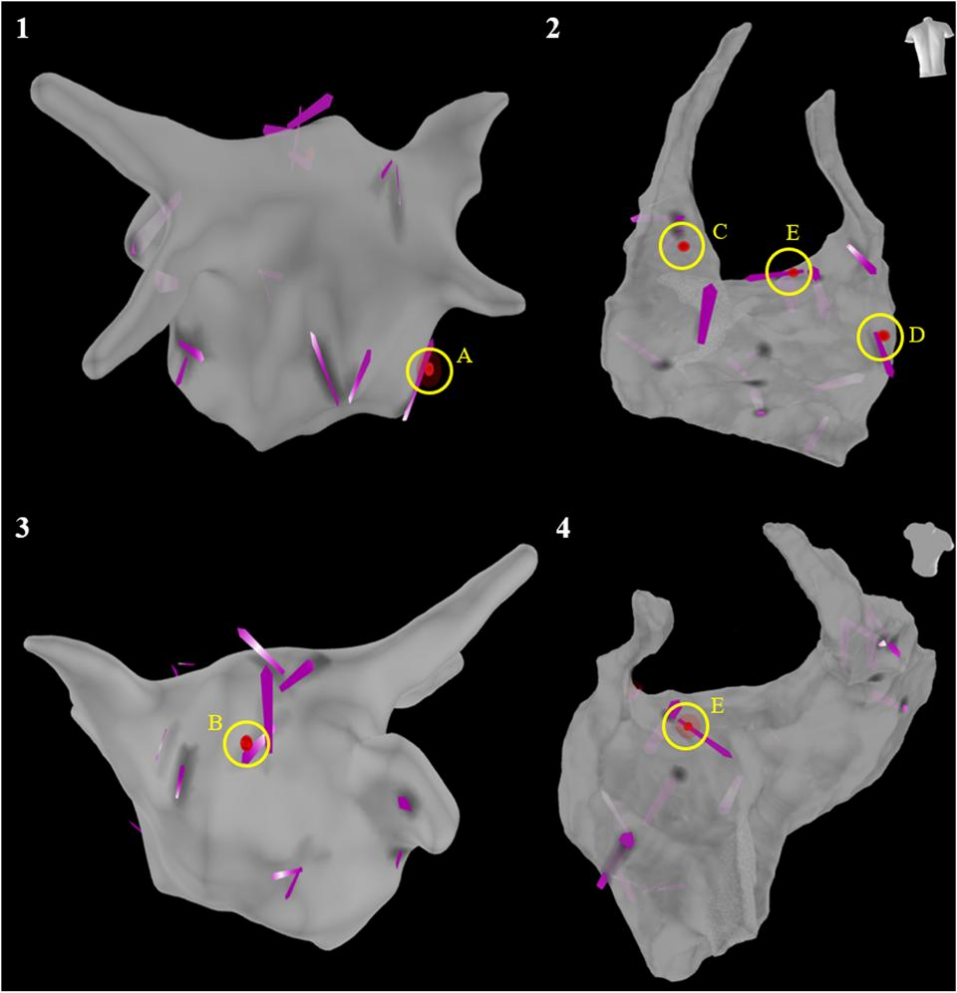
STAR Apollo maps of the left and right atria (1, PA view of the left atrium; 2, PA view of the right atrium; 3, AP roof view of the left atrium; 4, AP roof view of the right atrium) showing early sites of activation (dots) and repetitive patterns of activation (arrows).

Twelve acquisitions were made in the left atrium (LA) and 19 in the right atrium (RA) and PSLVC. The left atrium exhibited low voltages throughout, consistent with previous extensive ablation. A total of five ESAs (two in the LA, two in the RA, and one in the LPSVC) were identified, all of which had corresponding RPA STARburst patterns (*Figure 2*). There was one additional RPA STARburst on the lateral isthmus.

**Figure 3:**
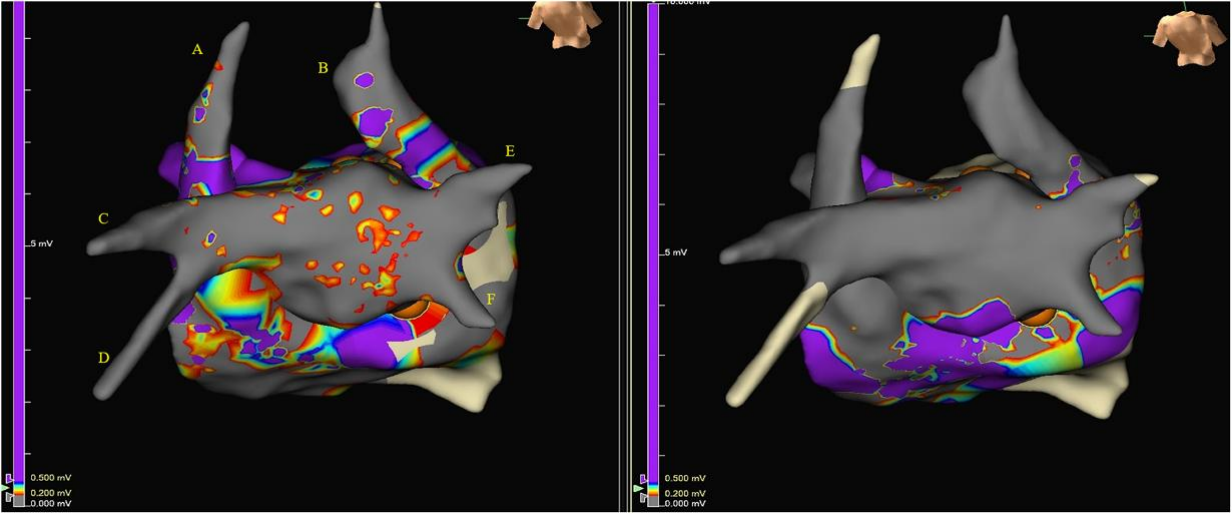
Pre-ablation and post-ablation voltage maps of both atria (PA view). (*A*) Persistent left superior vena cava, (*B*) superior vena cava, (*C*) left superior pulmonary vein, (*D*) left inferior pulmonary vein, (*E*) right superior pulmonary vein, and (*F*) right inferior pulmonary vein.

The right superior pulmonary vein (RSPV) still had residual signals; other pulmonary veins were silent. A voltage analysis map (*Figure 3*) showed widespread areas of high-frequency activation. The ablation strategy aimed for re-isolation of the RSPV followed by targeting of ESAs and STARburst RPAs.

### Ablation

Following pulmonary vein isolation, ESA sites in the LA were targeted with clustered pulsed field lesions; the patient had previously undergone an index cryoablation procedure and a redo procedure with radiofrequency energy, so surrounding structures would be less susceptible to damage from PFA energy especially if posterior wall/structure ablation was required. A pentaspline ablation catheter was used in the ‘flower’ configuration (Farawave™, Boston Scientific, MN). Early sites of activation were sequentially targeted in descending rank of frequency of repetition, with a site on the LA floor initially targeted (Site A, *Figure 2*). Atrial fibrillation terminated to sinus rhythm during ablation along LA roof (Site B, *Figure 2*). Atrial fibrillation was reinitiated using burst pacing with an isoproterenol infusion.

Attention then turned to the RA, with sequential targeting of ESAs (Sites C and D, *Figure 2*); once again, AF terminated at ablation on these sites and was then reinduced. Following ablation on an ESA on the RA septum (Site E, *Figure 2*), AF was no longer inducible; rather, burst pacing induced an atrial tachycardia with a cycle length of 270 ms. Entrainment appeared to confirm peri-mitral circuit; the tachycardia cycle length increased by 70 ms but did not terminate when PFA was applied to the lateral mitral annulus. A single RPA STARburst area within the PLSVC remained, and on targeting here the arrhythmia terminated; IV nitroglycerin was administered during ablation within the PLSVC to prevent coronary vasospasm. Voltage mapping confirmed electrical isolation (*Figure 3*). Notably, this final site of ablation mirrored a site identified in the LA, presumably indicating an electrical connection between these two chambers that was critical in supporting continuation of the arrhythmia.

No further arrhythmia could be induced despite extensive burst pacing with high-dose isoproterenol. Block across the lateral line was confirmed with differential pacing after systemic 30 mg adenosine challenge.

The procedure time was 91 min. There were no immediate complications from the procedure, and the patient was discharged home the same day. The patient remains arrhythmia free at 6-month clinical follow-up.

## Discussion

This is the first reported use of STAR Apollo Mapping in a patient with congenital abnormalities contributing to AF.

Broadly speaking, there are two major ways in which PLSVC poses challenges during catheter ablation procedures: identification of sites of origin of arrhythmia and logistical difficulties due to anatomical complexities. This case highlights how novel mapping techniques and PFA can be utilized to provide alternative solutions in these key areas and improve outcomes for complex patients where traditional methods fall short. STAR Mapping has undergone extensive validation, with electrogram timing and wavefront vector validation being undertaken by comparison with expert physician manual analysis. Identification of activation sequences in cell culture electrode arrays as well as in clinical pacing, clinical AT, and AF cases was undertaken in earlier studies.

Identifying target substrates for ablation is particularly challenging in patients undergoing a redo procedure. In a case such as this, where the patient had already undergone three previous ablations and showed minimal pulmonary vein (PV) involvement, identification of suitable ablation targets would be highly empirical. The use of advanced ‘AI’-based electrogram analysis technology in this case provided a specific guidance tool that permitted a targeted approach. Despite PVI being the mainstay strategy for catheter ablation of AF, PVI alone does not seem adequate in treating AF in PLSVC, with a high rate of recurrence despite persistent PV isolation.^13^ Pulsed field ablation has potential to reduce collateral damage to peri-atrial structures, including the oesophagus, which is especially useful when posed with unpredictable anatomical variances such as those seen in PLSVC.^14^ Although empiric right-sided SVC isolation alone can be advocated, in our patient, PFA application specifically to ESA and RPA STARburst sites resulted in the termination of AF that could not be reinduced, with no immediate complications. This implies that targeting mapped driver sites is a valid strategy facilitated by computational interpretation of electrograms, in this case the STAR Apollo Mapping system. The total procedure time was significantly lower than the mean procedure time of 151 min in a case series from 2022 of 20 PLSVC patients undergoing catheter ablation.^15^ These outcomes support the combination of PFA with STAR Apollo Mapping as a strategy for such complex catheter ablation procedures in AF.

## Conclusion

The successful termination of persistent AF in a patient with PLSVC using PFA guided by three-dimensional EnSite X mapping and STAR Apollo technology highlights the utility of novel methods of identifying arrhythmogenic origins in AF, especially in complex patients. These technologies have great potential to improve outcomes in PLSVC; further research is needed to evaluate them in greater depth.

## Lead author biography


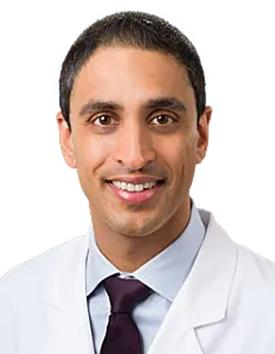


Dr Aaditya Vora is a regional leader in the field of electrophysiology and a primary investigator in both completed and ongoing clinical trials. He is passionate about improving his patients’ lives, driving him to stay at the forefront of medical technology, ensuring they benefit from the latest advancements. He places great importance on the role of being an educator for both his patients and colleagues alike. In addition, Dr Vora serves as the cardiac consultant for the Jacksonville Jaguars.

**Consent:** The authors confirm that written consent for submission and publication of this case report including images and associated text has been obtained the patient, in line with the COPE guidelines.

**Funding:** No funding was received for this research.

## Data Availability

The data underlying this article are available in the article.
